# Supplementary material for: BMP7 functions predominantly as a heterodimer with BMP2 or BMP4 during mammalian embryogenesis
Source: eLife. 2019 Sep 30;8:e48872. doi: 10.7554/eLife.48872 (PMC6785266; doi:10.7554/eLife.48872)
Supplement: Supplementary file 1. — Numbers and percent of animal of each genotype at P28. [file elife-48872-supp1.docx]

**Supplementary File 1. Progeny from *Bmp7^R-GFlag/+^* and *Bmp7^+/+^* intercrosses**

| Age | *Bmp7^+/+^* | *Bmp7^R-GFlag/+^* | Total |
| --- | --- | --- | --- |
| P28 | 155 (51%) | 151 (49%) | 306 |

Data are presented as number (percent).
