# Supplementary material for: BMP7 functions predominantly as a heterodimer with BMP2 or BMP4 during mammalian embryogenesis
Source: eLife. 2019 Sep 30;8:e48872. doi: 10.7554/eLife.48872 (PMC6785266; doi:10.7554/eLife.48872)
Supplement: Supplementary file 2. — (A) Progeny from Bmp7Flag/+ and Bmp2-/+ intercrosses. (B) Progeny from Bmp7Flag/+ and Bmp4-/+ intercrosses. (C) Progeny from Bmp7Flag/+ and Bmp7-/+ intercrosses. [file elife-48872-supp2.docx]

**Supplementary File 2A. Progeny from *Bmp7^Flag/+^* and *Bmp2^-/+^* intercrosses**

| **Age** | **Wildtype** | ***Bmp2^-/+^*** | ***Bmp7^Flag/+^*** | ***Bmp2^-/+^;Bmp7^Flag^*** | **Total** |
| --- | --- | --- | --- | --- | --- |
| P28 | 5 (21%) | 6 (25%) | 33 (29%) | 5 (21%) | 24 |

Data are presented as number (percent).

**Supplementary File 2B. Progeny from *Bmp7^Flag/+^* and *Bmp4^-/+^* intercrosses**

| **Age** | **Wildtype** | ***Bmp4^-/+^*** | ***Bmp7^Flag/+^*** | ***Bmp4^-/+^;Bmp7^Flag^*** | **Total** |
| --- | --- | --- | --- | --- | --- |
| P28 | 4 (22%) | 4 (22%) | 5 (28%) | 5 (28%) | 18 |

Data are presented as number (percent).

**Supplementary File 2C. Progeny from *Bmp7^Flag/+^* and *Bmp7^-/+^* intercrosses**

| **Age** | **Wildtype** | ***Bmp7^-/+^*** | ***Bmp7^Flag/+^*** | ***Bmp7^-/+^;Bmp7^Flag^*** | **Total** |
| --- | --- | --- | --- | --- | --- |
| P28 | 5 (29%) | 4 (23%) | 3 (18%) | 5 (29%) | 17 |

Data are presented as number (percent).
