# Supplementary material for: BMP7 functions predominantly as a heterodimer with BMP2 or BMP4 during mammalian embryogenesis
Source: eLife. 2019 Sep 30;8:e48872. doi: 10.7554/eLife.48872 (PMC6785266; doi:10.7554/eLife.48872)
Supplement: Transparent reporting form [file elife-48872-transrepform.docx]

***eLife’s* transparent reporting form**

We encourage authors to provide detailed information *within their submission* to facilitate the interpretation and replication of experiments. Authors can upload supporting documentation to indicate the use of appropriate reporting guidelines for health-related research (see [EQUATOR Network](http://www.equator-network.org/%20)), life science research (see the [BioSharing Information Resource](https://biosharing.org/)), or the [ARRIVE guidelines](http://www.plosbiology.org/article/info:doi/10.1371/journal.pbio.1000412) for reporting work involving animal research. Where applicable, authors should refer to any relevant reporting standards documents in this form.

If you have any questions, please consult our Journal Policies and/or contact us: [editorial@elifesciences.org](mailto:editorial@elifesciences.org).

**Sample-size estimation**

- You should state whether an appropriate sample size was computed when the study was being designed
- You should state the statistical method of sample size computation and any required assumptions
- If no explicit power analysis was used, you should describe how you decided what sample (replicate) size (number) to use

Please outline where this information can be found within the submission (e.g., sections or figure legends), or explain why this information doesn’t apply to your submission:

Analysis of animal survival and phenotyping. We did not perform a power analysis in advance because our preliminary data showed that that 0% of experimental animals (homozygous mutant) were detected at E12.5-P0, and that 100% of homozygous mutant animals were grossly abnormal by E10.5-11.5 (Table 1C). A similar finding was documented for compound mutants (Table 2). Based on this information, we minimized the number of animals used by intercrossing mice and collecting sufficient embryos at each stage to give a sample size that allowed us to collect a minimum of 3 (generally 10-15) wild type and mutant embryos to serve as biological replicates for all analyses that were conducted. Because the phenotypes are 100% penetrant, this gave sufficient power for statistical analysis of survival by Chi Square analysis, and of phenotypic defects.

**Replicates**

- You should report how often each experiment was performed
- You should include a definition of biological versus technical replication
- The data obtained should be provided and sufficient information should be provided to indicate the number of independent biological and/or technical replicates
- If you encountered any outliers, you should describe how these were handled
- Criteria for exclusion/inclusion of data should be clearly stated
- High-throughput sequence data should be uploaded before submission, with a private link for reviewers provided (these are available from both GEO and ArrayExpress)

Please outline where this information can be found within the submission (e.g., sections or figure legends), or explain why this information doesn’t apply to your submission:

In situ hybridization, immunostaining and ßgal staining experiments involved analysis of 1-5 wild type or homozygous mutant littermates (biological replicates) per experiment and analysis was repeated in three technical replicates. The total number (*n*) of embryos of each genotype that were analyzed is indicated in the text in the results section. For Western analysis of pSmad levels (Fig. 4) and co-IP analysis of heterodimer formation (Fig. 6) material was collected from three biological replicates and analyzed in three technical replicates. This is indicated in the Figure legend.

**Statistical reporting**

- Statistical analysis methods should be described and justified
- Raw data should be presented in figures whenever informative to do so (typically when N per group is less than 10)
- For each experiment, you should identify the statistical tests used, exact values of N, definitions of center, methods of multiple test correction, and dispersion and precision measures (e.g., mean, median, SD, SEM, confidence intervals; and, for the major substantive results, a measure of effect size (e.g., Pearson's r, Cohen's d)
- Report exact p-values wherever possible alongside the summary statistics and 95% confidence intervals. These should be reported for all key questions and not only when the p-value is less than 0.05.

Please outline where this information can be found within the submission (e.g., sections or figure legends), or explain why this information doesn’t apply to your submission:

Based on the nature of these studies, the only statistical analysis is a simple 2-tailed T test for Figure 3O.

(For large datasets, or papers with a very large number of statistical tests, you may upload a single table file with tests, Ns, etc., with reference to sections in the manuscript.)

**Group allocation**

- Indicate how samples were allocated into experimental groups (in the case of clinical studies, please specify allocation to treatment method); if randomization was used, please also state if restricted randomization was applied
- Indicate if masking was used during group allocation, data collection and/or data analysis

Please outline where this information can be found within the submission (e.g., sections or figure legends), or explain why this information doesn’t apply to your submission:

Embryos were scored for morphology prior to genotyping. Researchers were also blinded to genotype prior to photodocumentation and scoring for immunostaining and ß gal staining. This is described in the methods.

**Additional data files (“source data”)**

- We encourage you to upload relevant additional data files, such as numerical data that are represented as a graph in a figure, or as a summary table
- Where provided, these should be in the most useful format, and they can be uploaded as “Source data” files linked to a main figure or table
- Include model definition files including the full list of parameters used
- Include code used for data analysis (e.g., R, MatLab)
- Avoid stating that data files are “available upon request”

Please indicate the figures or tables for which source data files have been provided:

Not applicable.
